# Staying Home, Staying Alive: Campus Food Pantry Student Clients’ Experiences During the COVID-19 Pandemic

**DOI:** 10.1177/19367244211035671

**Published:** 2022-03

**Authors:** Elmira Jangjou

**Affiliations:** 1 The University of Iowa, Iowa City, USA

**Keywords:** food insecurity, higher education, coping, resilience, pandemic

## Abstract

In response to students’ food insecurity, a number of colleges and universities have taken action and established campus food pantries as part of their intervention plans. However, many of these pantries ceased operation due to COVID-19 campus shutdowns. The purpose of this study is to illustrate the short-term impacts of the COVID-19 pandemic on postsecondary students, who use a university-provided food pantry. Drawing from semi-structured interviews with 12 participants, the thematic analysis explored the initial coping strategies these students used to endure the pandemic. Findings revealed that many students experienced the immediate effects of the pandemic in the form of income loss, self-isolation, anxiety, and appetite change. Although the pandemic interrupted these students’ journeys to continue their studies and become independent in various ways, the affected students implemented various coping strategies, including seeking help from family or friends, using available resources, cooking at home, and even trying to save money. However, considering that the targeted population in this study was already at risk because of their basic needs insecurity, these postsecondary students require extra attention from their higher education institutions in the case of emergencies, such as a global pandemic. In addition to its timely and relevant findings, this study provides important avenues for future research and intervention efforts.

## Introduction

Higher education represents a period of transition from adolescence to adulthood. Although this period can provide individuals with opportunities for identity exploration and positive experiences, it can also be challenging for those from vulnerable populations (Arnett 2000, 2007). As Maslow’s human development theory suggests, basic needs like food must be satisfied to allow individuals to pursue higher level skills (Maslow 1943). When a student’s basic needs are not met (in this case, with sufficient nourishment), it becomes more difficult for them to successfully meet the demands of higher education, specifically those that require higher level skills (Broton 2017; Farahbakhsh et al. 2017; Goldrick-Rab et al. 2018; Maroto, Snelling, and Linck 2015; Phillips, McDaniel, and Croft 2018). Therefore, the prevalence of food insecurity among postsecondary students remains a vital issue in higher education (Meza et al. 2019).

According to various studies in higher education, 14 to 59 percent of postsecondary students experience food insecurity (Henry 2017), which is higher than the 2018 national average of 11.1 percent for general U.S. households (Coleman-Jensen et al. 2019). The United States Department of Agriculture (USDA) defines food security as “access by all people at all times to enough food for an active, healthy life,” which is “one of several conditions necessary for a population to be healthy and well nourished” (Coleman-Jensen et al. 2019:2). Although millions of students today can access higher education, the task of balancing college expenses remains difficult for many. For some students from low-income backgrounds, attending college can often mean sacrificing their basic needs, such as food. Additionally, certain students may face situational poverty for the first time as “part of the new economics of college” (Goldrick-Rab 2016:128). For many emerging adults, who are in the stage between adolescence and young adulthood, going to college is the first step toward economic independence. There is frequently the cultural and familial expectation that in addition to managing their academic life, they will move out of their parents’ households and gradually become independent from their parents by “accepting responsibility for [themselves], making independent decisions, and becoming financially independent” (Arnett 2015:49). However, this transition to independence can be challenging for many individuals and might even put them at a higher risk of food insecurity.

Due to complex and limiting eligibility criteria and a general lack of information, few students are able to rely on federally funded safety-net programs dedicated to aiding those who experience food insecurity in the United States; such programs include the Supplemental Nutrition Assistance Program (SNAP), and the Special Supplemental Nutrition Program for Women, Infants, and Children (WIC) (Broton and Goldrick-Rab 2018; Broton, Weaver, and Mai 2018; El Zein et al. 2018; Goldrick-Rab et al. 2019; Henry 2020). As international students are ineligible for SNAP benefits, this restriction makes food pantries an even greater resource for them. Even for U.S. citizens, however, it is difficult for college students to qualify for and take advantage of these benefits (Broton and Cady 2020; El Zein et al. 2018; Goldrick-Rab 2016). As such, unlike federally funded resources, seeking help from family and friends is one of the common coping strategies among postsecondary students (Broton et al. 2018). This again puts some international students at a disadvantage, as it is more difficult for them to access their family resources from a distance.

Generally associated with community and social service programs, food pantries are an essential response to ongoing food insecurity issues (Mabli et al. 2010). In this vein, many colleges and universities have established campus food pantries. This expansion is in response to the growing awareness of the basic needs of the diverse student population that frequents institutions of higher education (Broton and Cady 2020; Cady and White 2018; Gupton, Trost, and Collins 2018; Reppond et al. 2018). In a qualitative study, Henry (2020) concludes that “tipping points” are one of the common reasons that students who have become food insecure decide to reach out to on-campus food pantries (p. 28). These tipping points can include, among others, “changes in living situations, reduction in work, . . . [and] medical crises” that result in unstable finances (p.29). From this perspective, the current COVID-19 pandemic can certainly be categorized as a tipping point that has endangered the food security of students.

In response to the COVID-19 pandemic, many universities closed their campuses (Foresman 2020), effectively cutting students off from campus food pantries (Goldrick-Rab et al. 2020). It is worth noting that these closures occurred when many students and their families were facing unemployment (Wolfers 2020). The Hope Center for College, Community, and Justice, which defines itself as “a nonprofit research center focused on rethinking and restructuring higher education and social policies, practices, and resources to create opportunities for all students to complete college degrees” (Broton and Cady 2020:282), conducted an online survey with 38,602 respondents between April 20, 2020 and May 15, 2020. According to their findings, about 60 percent of students reported basic needs insecurity during the pandemic, and 64 percent of students who were employed before the pandemic reported job insecurity (Goldrick-Rab et al. 2020). These results concerning the basic needs of postsecondary students during the COVID-19 pandemic are alarming and therefore demand further investigation.

## Theoretical Framework

In an attempt to further understand food insecurity conditions in higher education, this study was guided by the resilience developmental theory (Masten 2001). The basic premise of this theory is that, regardless of any negative experiences individuals have had with significant risk exposure in the past, they tend to demonstrate resilience when they maintain a positive adaptation, making it possible for them to cope successfully with any challenges they face (Luthar 2006; Masten 2015; Rutter 2012). In other words, individuals’ personal characteristics (e.g., autonomy and problem-solving skills) and their available social supports (e.g., parental support) can moderate the harmful effects of adversity and promote positive results regardless of existing risks (Benard 1991; DuMont, Ehrhard-Dietzel, and Kirkland 2012; Greene 2002; Masten 1994).

Scholars in the field of higher education have also implemented the resilience theory, suggesting that a high level of resilience can predict students’ adjustment and adaptation to college life (Banyard and Cantor 2004; Fassig 2004). Additionally, student resilience and self-efficacy in conjunction with their social capital play a critical role in “high-risk” postsecondary students’ retention and graduation (Avery and Daly 2010:46). The resilience theory can therefore be useful when exploring how postsecondary students already coping with barriers to accessing sufficient food have been influenced by the COVID-19 pandemic and the subsequent loss of access to campus food pantries.

## Current Study

Despite the recent rise in literature about food insecurity within higher education, this field of study remains largely under-researched. In particular, there is a great deal to be discovered about university/college food pantries and students’ experiences utilizing this service. Although food pantries are a distinct and accessible solution to the issue of food insecurity among postsecondary students, this may not be the case in certain emergency situations, including nation-wide pandemics. The initial purpose of this study was to assess the overall experiences that clients had with on-campus food pantries. However, due to the implications of COVID-19 pandemic, the study’s purpose underwent several changes. When the university shut down in March 2020, the food pantry also ceased operations. The students that I continued to interview frequently cited the effects of the pandemic. Therefore, I began asking follow-up questions about how recent events have influenced their lives specifically. For this reason, the primary goal of the current study is to report several short-term impacts of the COVID-19 pandemic on the student clients of a university food pantry and the initial coping strategies that the students used to endure the effects of the pandemic. The central research question guiding this study is: *How did the COVID-19 pandemic affect campus food pantry student clients in the early days of this pandemic?* The secondary research question arose while analyzing the interview data. Students shared their coping strategies while explaining how they were affected by the pandemic. Therefore, the second research question that this study tries to answer is: *How did these students cope with the immediate changes that happened in their lives due to the pandemic?*

This study aims to raise awareness about the specific needs of a diverse group of postsecondary students during this particular emergency. The findings and related implications of this study can be used to inform colleges and universities of the inequities within higher education, relevant to the pandemic and related economic crises. By adding student voices and perspectives, this study intends to help administrators and other decision-makers in higher education institutions, who may face similar challenges at their campuses, make informed decisions during similar emergencies to ensure that the students’ basic needs are met.

## Method

### Sample

The participants were selected using a purposeful sampling method after obtaining approval from the Institutional Review Board (IRB). The food pantry staff sent invitation emails to all students who had visited the food pantry from Summer 2016 to Spring 2020. Invitation emails were sent in two rounds. After the first attempt, six students expressed their willingness to participate in the study. Monetary incentives (a gift card worth $20 for each interviewee) were offered in the second round to attract more students. Nine more students volunteered to participate in the study. Interested participants contacted me and scheduled meetings. Participants were sent informed consent forms. They had an opportunity to review the form and ask questions about the study prior to agreeing (or not agreeing) to consent to participation.

Fifteen students participated in this study over a 2-month period (March 2020 to May 2020). This study uses interview data from 12 participants who discussed their experiences during the COVID-19 pandemic. Three students were excluded from this study for the following reasons: Two of the first interviewed students did not explicitly mention the pandemic. Therefore, there was no interview data that could answer the research questions of the current study. The other interviewee was disqualified since she had graduated before the pandemic started. Table 1 presents the self-reported information of all 12 student clients. Pseudonyms are used to de-identify the participants. The majority of students were female (83 percent), self-identified as White (42 percent), and enrolled as graduate students (58 percent). Most of the participants were full-time (92 percent) and in-state students (75 percent). Five participants (42 percent) self-identified as first-generation students. The majority of the interviewees (75 percent) reported having part-time jobs.

**Table 1.** Demographic Information of the Participants.

Pseudonym	Age	Sex	Ethnicity	Student status	Full-time student	First-gen student	Residency status	Employment status	Year of study
Sam	21	Female	Caucasian/White	Undergraduate	Yes	No	In-State	Full-time	Third
Jaime	26	Female	Caucasian/White	Graduate	Yes	No	In-State	Part-time	First
Colette	23	Female	Caucasian/White	Graduate	Yes	No	In-State	Part-time	First
Dave	23	Male	Caucasian/White	Undergraduate	Yes	Yes	In-State	Part-time	Third
Sal	22	Female	African American/Black	Undergraduate	Yes	No	In-State	Part-time	Fourth
Jay	24	Male	Asian	Graduate	Yes	No	In-State	Part-time	First
Selena	23	Female	African American/Black	Undergraduate	No	Yes	In-State	Part-time	Third
Faith	22	Female	Caucasian/White	Undergraduate	Yes	Yes	In-State	Part-time	Fourth
C	29	Female	Asian	Graduate	Yes	Yes	International	Full-time	Fifth
Maria	23	Female	African American/Black	Graduate	Yes	No	In-State	Part-time	Second
Bubly	30	Female	Asian	Graduate	Yes	No	International	Full-time	Second
Kat	28	Female	Asian	Graduate	Yes	Yes	International	Part-time	Second

### Procedure

Data were collected via semi-structured interviews. Interviewees were also asked to fill out a demographic information form concerning age, sex, ethnicity, student status, residency status, and employment status as part of the interview. Interviews were held over the phone or through Zoom Video Conferencing due to the unexpected COVID-19 pandemic. The semi-structure interviews followed an interview guide to ensure consistency among interviews. The guide included open-ended questions about the students’ experiences with the university food pantry in general. During the interviews, if a participant mentioned the pandemic, they were also asked follow-up questions about how the COVID-19 pandemic has influenced their lives. Each interview was audio taped with the interviewee’s permission and later transcribed verbatim (using Rev.com.) for data analysis.

### Data Analysis

The current study uses a qualitative research design to understand the lived experiences of postsecondary students during the time of COVID-19. Qualitative research is a common research design to study food insecurity in higher education (Henry 2017, 2020; Meza et al. 2019; Stebleton, Lee, and Diamond 2020; Watson et al. 2017). This research method allows scholars to capture students’ voices and perspectives and better understand their experiences with food insecurity (Mulligan and Brunson 2017). To analyze the data, thematic analysis was used to generate the codes and themes of the interview in order to provide insight into the participants’ immediate experiences with the COVID-19 pandemic. Thematic analysis allows researchers to make sense of experiences by connecting the narratives and finding common data categories (Braun and Clarke 2012). I adopted Nowell et al. (2017) suggestion on the six phases to conduct a trustworthy thematic analysis. In the first phase, I familiarized myself with the data by going through the interview transcripts repeatedly and determined what data pertained to students’ experiences related to COVID-19. The second phase was generating initial codes in Dedoose. Phases three to five, searching for themes, reviewing themes, and defining and naming themes, are documented in Microsoft Word files. In these cycles of coding, pattern and axial coding enabled me to combine the initial codes and generate themes (Saldaña 2015). Coding and themes concerning the research question of this study were discussed during the research team meetings. Although these meetings were generally about the broader research on students’ experiences with the campus food pantry (discussed earlier in the *Current Study* section), a portion of this meeting was spent discussing the themes about the pandemic and reaching team consensus on these themes. I also took a personal journal to document these ideas. The final phase was producing a report of the findings. These six phases worked together to establish trustworthiness during the thematic analysis (Nowell et al. 2017).

## Results

The thematic analysis revealed three main categories for this study: short-term readiness, immediate impacts, and coping strategies. The first theme centers upon the students who did not think the pandemic influenced their lives dramatically. The second theme describes the immediate effects that the pandemic had on some postsecondary students. These impacts are broken down into three domains of financial, structural, and mental health. Finally, the third theme explains the students’ coping strategies, which consists of three secondary themes: seeking help, making use of available resources, and stay home, save money.

### Short-Term Readiness

The interviews were conducted during the uncertain early days of the COVID-19 pandemic, when it was still unclear for the students whether the situation would have long-term effects and how long the campus food pantry would stay closed. Some interviewees stated that they were “ready” for the pandemic because of their recent visit to the food pantry and the accumulated food in their homes, hoping everything would return to normal soon. When I asked Colette, a graduate student, how the pandemic affected her access to food and her food pantry usage, she said,

I feel like I was pretty lucky in that I had accumulated enough food that I didn’t feel like I was in an immediate need for anything. So, when the food pantry closed, I felt pretty comfortable. And then just having the Walmart nearby, that’s pretty nice. I don’t feel like I’m in a really tight position, financially or food-wise because I had the food pantry there and I wasn’t going through everything I was getting from food pantry within one week. So, I still have some beans left over and there’s a lot that I can do there. So yeah, I feel okay. Of course it’s stressful, just the nature of the situation, but I’m not in a bad place.

Selena, another student who mentioned that she was thus far unaffected by the pandemic, stated, “I haven’t noticed it affecting me. I’ve been able to receive more help of EBT, but personally, I don’t feel affected with food when it comes to the pandemic.” Having access to a federal food assistance program helped Selena ensure that she still had access to food while the food pantry was shut down. When I asked C, an international student who was close to graduation, how the pandemic had affected her eating schedule, she responded,

Mostly I eat two meals a day. Now I have a car so . . . After I purchased my car, the time we were going to the food pantry was reduced. So, now I have enough food at home, and I think I will not purchase much food now because I will leave [the city] next month. I want to make sure that I eat up all the food before I leave.

As a 29-year-old student, C was in the very last stage of completing her graduate degree. She resolved to alleviate her transportation problem by getting a car, as not having a vehicle was limiting her access to grocery stores during the final year of her graduate studies in the United States. For C, having a car and knowing that she would soon be leaving the university decreased her vulnerability to the food pantry’s shutdown.

### Immediate Impacts

#### Financial

Although some students did not immediately notice the pandemic’s impact on their lives, others reported experiencing the early consequences of the pandemic. A few students, for instance, faced an immediate loss of income due to a job loss. Dave explained, “A lot of my food before, it came from me, myself and my part-time job and you know, that has been immediately cut off. You know, I, I basically have no income now.” Dave’s job at a restaurant was not only his source of income but also “where [he] would get some of [his] food.” Faith also faced financial difficulties as a result of the pandemic, stating,

My household is myself and my fiancé and now my fiancé’s on unemployment, so he’s making less money, but we still have to pay rent and electric bill and for gas and stuff. So now we basically don’t have any extra income. It just goes right to bills. So, we’re using up our savings to buy it. We had some money saved up before this happened and now it’s depleting because we’re using that to buy groceries and stuff.

#### Structural

Some of the effects of the COVID-19 pandemic were structural in nature. The closure of the campus food pantry was one of these structural impacts. Sam, for instance, recently decided to begin utilizing the food pantry more regularly because of her unstable financial situation. However, she notes that she “didn’t really get the opportunity to really start using it” due to the food pantry closure. Due to the university’s transition to online/remote learning, taking online classes was another immediate impact of the pandemic on the students’ lives. Having lost his part-time job at a restaurant because of the pandemic, Dave said, “I’m just, bunkering down and making it through quarantine, and taking my classes and staying busy with online work.” This transition also enabled a few students to move into their parents or friends’ house, which gave them emotional and economic support during the stressful days of the pandemic.

#### Mental health

Students also reported changes to their behavior as a result of the pandemic. Such behavior included self-isolating, going out less, experiencing anxiety, and eating less. Jaime, who was “trying to avoid going out too much” because of her chronic illness, decided to “self-isolate” during the pandemic. With regard to her appetite change, she said, “Right now. I’m just. I know I’m anxious so I’m not eating as much which is a little silly but true.” She was not the only one to report changes in eating habits. Indeed, Faith and Maria also began to eat fewer meals during the pandemic. Bubly, who was unaware of the food pantry closure, also made some adjustments in her life due to the pandemic, stating,

Actually, I just stopped going out as usual. I just go out to buy groceries and then, because I only eat one meal in a day, so it’s actually quite easy for me. Because I need, I just need a little, little amount of food, and then that’s enough for me for the day. So, I go to the supermarket once, the food probably can last about two weeks. So, and I didn’t go to food pantry. I try to avoid connecting, interacting with people.

### Coping Strategies

#### Seeking help

Students also described some of the social ties that were helpful when facing the uncertainty of the early days of the pandemic. Transitioning to parents’ or friends’ houses was one of the immediate ways that the pandemic impacted their lives. Although, this could be categorized as an additional disruption to the students’ lives, this change also guaranteed that these individuals had access to food. Kat, who is an international student, explained how she reached out to her friends and said, “I now live with my friends on the farm. So, I’m just eating their food. I don’t think I experience much difficulties now without food, because of the help of my friends.” Jay, Dave, and Maria also talked about their need for parental support. Jay stated, “Now that I’m home, I’m not having to buy my own food. So, in that regard, I don’t worry about food anymore.” Dave, who lost his part-time job in a restaurant, relied on his mother’s financial support. Maria, however, shared her disappointment with the food pantry closure by explaining,

Luckily, I’ve been getting money from the school, but if I hadn’t had been getting money from this school, then I would probably have to go back to my parents’ house to be able to get food. And that’s why I was pretty sad that the pantry was not open during the pandemic, because that would have been a perfect resource during this time.

Maria’s statement clarifies her own vulnerability to income loss. Because her supplementary source of food (in this case, the food pantry) was no longer an option, she had to decide whether she needed to return to her parents’ home to ensure that had access to food.

#### Making use of available resources

Due to the closure of the campus food pantry, students were forced to figure out what other resources were available to them. Students mentioned going to the grocery store, which resulted in “spending more money on food.” Jaime and Selena talked about “stretching” their SNAP/EBT benefits during this unprecedented emergency. Unlike the campus food pantry, public food pantries remained open during the pandemic, providing another resource for the students to meet their basic needs. Jaime, for instance, mentioned going to a public food pantry close to the university campus a few times during the pandemic; however, her chronic illness prevented her from continuing to take advantage of it because

when you go there to pick up a premade box, there are still like a whole bunch of people in a really long line and I was just like around too many people still and exposed to too many germs.

For Dave, his tax return was “another thing helping keep [him] alive.”

#### Stay home, save money

Attending classes online and staying home helped Jamie and Sal save money and cut some of their expenses. Scrutinizing the smallest savings, Jamie explained how having more time to make breakfast could help her to spend less on food:

I can make oatmeal in the morning for breakfast instead of having to have something I can like a granola bar I can grab and run out the door with and I feel like oatmeal is like 10 cents a bowl or as a granola bar is like 50 cents a bar, so that is a big change in breakfast cost.

Sal saw taking online classes as an opportunity to save money, sharing,

It makes going to the grocery store less. The trips will be less. But at the same time, it helps in the budget because I don’t have to go anywhere, and classes are at home. I guess that’s an advantage of having online classes. So, I just stay at home, so I’ll be cooking and then I haven’t class, so it’s not too bad.

## Discussion

This research aimed to investigate the short-term effects of the COVID-19 pandemic on postsecondary students who use an on-campus food pantry at a Midwestern public research university. The initial coping strategies that the Students used to endure the effects of the lockdown were also assessed. Similar to previous studies, participants had diverse experiences with food insecurity, which confirms the subsequent need for a multi-faceted approach to promote food security in higher education (Broton et al. 2018). Depending on their unique situations and personal contexts, the students in this study faced numerous hardships. Although some students reported short-term readiness for the COVID-19 pandemic and the closure of the campus food pantry, the data also documented that several students faced financial, structural, and mental health effects. Some students and their family members faced an income loss, which was expected according to various reports about the COVID-19 pandemic (Goldrick-Rab et al. 2020; Wolfers 2020). According to the existing literature, financial difficulties motivate students to visit campus food pantries (Broton and Cady 2020; El Zein et al. 2018; Henry 2020). In response to the pandemic, many universities, including the university at which this study was conducted, closed their campuses (Foresman 2020) and their campus food pantries stopped operating. With the food pantry’s closure, grocery stores became the primary food resource for many students in this study, which resulted in spending more money on food and required cutting corners and making sacrifices. Students also reported self-isolating, going out less, experiencing anxiety, and eating less. These findings confirm my hypothesis that the COVID-19 pandemic, even in the early days, was a tipping point (Henry 2017, 2020).

To cope with the effects of the COVID-19 pandemic and their loss of access to the campus food pantry, students generally devised short-term solutions to address their nutritional needs. Some of them reached out to their family and friends, as a common coping strategy among postsecondary students (Broton et al. 2018). For some students, moving back into parents’ houses or moving in with someone else became a coping strategy during the pandemic as a way to ensure resources such as food. Although returning home is not uncommon among emerging adults, it can interrupt their transition to adulthood and financial independence (Arnett 2015). Moreover, previous research suggests that food-insecure students are at a high risk of experiencing housing insecurity too (Broton and Cady 2020; Goldrick-Rab et al. 2019; Henry 2020). My findings also confirm the existing literature that speaks to students’ lack of money and time as major barriers to food security (Broton et al. 2018; Henry 2020; Maynard et al. 2018). However, as a result of the COVID-19 pandemic, students had more time to cook at home, which helped them to save money as well. Stretching their SNAP benefits and changing their eating habits were some of the other coping strategies reported by the interviewees.

According to the findings of this qualitative study, the COVID-19 pandemic can be classified as an interruption in the lives of postsecondary students, challenging their food access. Coping strategies, including taking advantage of federally-funded safety net programs and seeking help from family and friends, were thus implemented to express resilience, maintain an optimistic viewpoint, and overall moderate the harmful effects of the pandemic (Benard 1991; DuMont et al. 2012; Greene 2002; Luthar 2006; Masten 1994, 2015; Rutter 2012). However, it is worth noting that many of these strategies were short-term in nature. The disturbing nature of these student responses highlights the importance of campus food pantries during emergencies like the COVID-19 pandemic. These findings further convey that higher education institutions must be held accountable for ensuring and facilitating student access to emergency aid packages. Colleges and universities must also provide sufficient information on federally funded safety-net programs like SNAP to ensure that the lives of vulnerable students are not crippled by unexpected emergencies (Broton and Goldrick-Rab 2018; Broton et al. 2018; El Zein et al. 2018; Goldrick-Rab et al. 2019; Henry 2020).

### Limitations

One of the main challenges of this study was to attract a diverse group of students to volunteer to participate in this study. This limitation stemmed from the purposeful sampling method that was used in this research. As such, it would not be possible to generalize the results to campus food pantry clients in general. Additionally, the emergence of the COVID-19 pandemic imposed limitations, too. Considering the closure of the university and how vulnerable the targeted population for this study was, it is possible that students in a more severe situation did not have internet access to receive the invitation emails, or they were too busy dealing with the challenges and uncertainty that the pandemic brought into their lives.

### Implications for Future Research and Practice

The findings of this study can be useful to all those interested in understanding and supporting the resilience of food insecure postsecondary students. Besides its timely and relevant findings, the current study provides important avenues for future research and intervention efforts. The targeted population in this study was already at risk because of their basic needs insecurity before the COVID-19 pandemic. The results suggest that future research would benefit from a more nuanced investigation of this global pandemic’s effects on not only students who were food pantry clients before the pandemic but also students who became food insecure as a result of this phenomenon. Additional research that further examines the long-term impacts of the COVID-19 pandemic on postsecondary students is also needed to deepen our understanding of how contextual factors may influence students’ experiences with food insecurity and their related coping strategies in the long run.

The diversity of experiences that students had with food insecurity underlines the necessity of a multi-faceted approach in higher education (Broton et al. 2018). Even though short-term solutions for students’ basic needs insecurity, including campus food pantries and emergency funds, are beneficial, more systematic and institutional changes are required. Moreover, “a cultural shift that sees financial, family, and health issues as integral to academics, recognizes poverty as a societal problem rather a personal failing, and unites college and community in partnerships to provide support” is needed to facilitate students’ graduation (Goldrick-Rab and Cady 2018:3). As the findings of this study indicate, students’ food and housing security, as well as their physical and mental health, all influence the student’s academic lives.

Universities should also be prepared to meet the needs of not only the students who relied on campus food pantries before the pandemic but also other students who are at risk of food insecurity due to the pandemic. The COVID-19 pandemic and closure of universities and campus food pantries highlight the importance of long-term strategies and improving access to the federally funded safety-net programs (such as SNAP and WIC) to address food insecurity among postsecondary students. Furthermore, it is important that those of us in higher education be aware that international students are not eligible for these benefits, even during emergencies. While those of us in higher education might not be able to immediately influence this policy, knowing that international students have even less options during this time can guide our institutional practices and outreach. The findings of this study provide valuable knowledge about the specific needs of a diverse group of postsecondary students during emergency situations that endanger their food security. While this study examined the experiences of students who were using the food pantry before the pandemic, it also highlights the precarious economic situation in which many students now find themselves. Therefore, the findings and related implications of this study can be used to inform leaders and decision makers in higher education institutions of the inequities within higher education, particularly as they relate to emergencies and related economic crises. Higher education institutions must be prepared to make informed decisions during crisis situations in order to ensure that the basic needs of their student population, including food and housing, are met, most notably by improving the services that on-campus food pantries provide and making it easier to access such facilities during emergencies. Finally, this study highlights the essential need for policies that embrace such long-term objectives as affordable higher education for all students, particularly those already challenged with food insecurity.

## Conclusion

The findings and discussion highlighted in this article point to the growing issue of food insecurity in higher education during the COVID-19 pandemic. Although the primary concern identified in this study involves postsecondary students who use an on-campus food pantry at a Midwestern public research university, the research also demonstrates the overarching impacts of this global pandemic and the broader issue of postsecondary students’ basic needs insecurity. In this regard, the case of a campus food pantry student clients and their coping strategies facing a global pandemic paves the way for expanded considerations of the role of campus food pantries in the lives of postsecondary students.
